# Recent Progress in Understanding Subtype Specific Regulation of NMDA Receptors by G Protein Coupled Receptors (GPCRs)

**DOI:** 10.3390/ijms15023003

**Published:** 2014-02-20

**Authors:** Kai Yang, Michael F. Jackson, John F. MacDonald

**Affiliations:** 1 Robarts Research Institute, Molecular Brain Research Group, University of Western Ontario, 100 Perth Drive, London, ON N6A 5K8, Canada; E-Mail: jfmacdonald@robarts.ca; 2 Department of Pharmacology & Therapeutics, University of Manitoba, Winnipeg, MB R3E 0T6, Canada; 3 Neuroscience Research Group, Kleysen Institute for Advanced Medicine, Health Sciences Centre, University of Manitoba, Winnipeg, MB R3E 3J7, Canada; 4 Department of Physiology and Pharmacology, University of Western Ontario, London, ON N6A 5K8, Canada

**Keywords:** NMDA receptor, G protein coupled receptor, protein kinase A, protein kinase C, cyclic AMP

## Abstract

G Protein Coupled Receptors (GPCRs) are the largest family of receptors whose ligands constitute nearly a third of prescription drugs in the market. They are widely involved in diverse physiological functions including learning and memory. NMDA receptors (NMDARs), which belong to the ionotropic glutamate receptor family, are likewise ubiquitously expressed in the central nervous system (CNS) and play a pivotal role in learning and memory. Despite its critical contribution to physiological and pathophysiological processes, few pharmacological interventions aimed directly at regulating NMDAR function have been developed to date. However, it is well established that NMDAR function is precisely regulated by cellular signalling cascades recruited downstream of G protein coupled receptor (GPCR) stimulation. Accordingly, the downstream regulation of NMDARs likely represents an important determinant of outcome following treatment with neuropsychiatric agents that target selected GPCRs. Importantly, the functional consequence of such regulation on NMDAR function varies, based not only on the identity of the GPCR, but also on the cell type in which relevant receptors are expressed. Indeed, the mechanisms responsible for regulating NMDARs by GPCRs involve numerous intracellular signalling molecules and regulatory proteins that vary from one cell type to another. In the present article, we highlight recent findings from studies that have uncovered novel mechanisms by which selected GPCRs regulate NMDAR function and consequently NMDAR-dependent plasticity.

## 1. The Introduction of G Protein Coupled Receptors (GPCRs)

GPCRs (G protein coupled receptors) are the largest family of transmembrane receptors and their clinical importance is evident by the fact that nearly a third of prescription drugs target these receptors [1]. GPCRs have a common structural motif that consists of seven transmembrane helices, in which the *N*-terminus is extracellular and the *C*-terminus is intracellular. When a GPCR is activated, its conformation changes and allows the receptor to interact with G proteins. The exchange of GDP for GTP dissociates Gα from Gβγ subunits, subsequently activating various intracellular effectors [2]. The activation of G proteins can be terminated by regulators of G protein signalling (RGS) proteins, resulting in the cessation of signalling pathways induced by GPCRs [3]. Of note, the ability of some GPCRs to signal independently of G proteins is being increasingly recognized [4].

GPCRs include three distinct families: A, B and C, based on their different amino acid sequences. Family A is the largest group including muscarinic acetycholine receptor, dopamine receptor and sphingosine 1-phosphate receptor. Family B has only 25 members, including PAC1 (pituitary adenylate cyclase activating peptide) receptor and VIP (vasoactive intestinal peptide) receptor. Family C is also relatively small and contains the metabotropic glutamate receptors (mGluRs) as well as some taste receptors and all family members have very large extracellular domain that mediate ligand binding and activation [5].The Gα subunit that couples with these receptors is also used to classify receptors. Four families are identified namely, Gαq, Gαs, Gαi/o and Gα_12/13_. The Gαq pathway activates phospholipase C beta (PLCβ) to produce inositol trisphosphate (IP_3_) and diacylglycerol (DAG). The Gαs pathway usually stimulates adenylate cyclase (AC) activity whereas the Gαi/o family inhibits it. In contrast, Gα_12/13_ stimulates Rho activity and induces cytoskeleton remodelling [6].

Collectively, GPCRs are widely involved in diverse physiological functions. They have an important influence on learning and memory as evidenced by impaired memory associated with the dysfunction of GPCRs. For example, genetic ablation of the muscarinic M1 receptor is associated with cognitive dysfunction [7]. In this capacity, GPCRs likely influence learning and memory by regulating excitatory synaptic transmission and plasticity. Specifically, NMDA receptors (NMDARs), which belong to the family of ionotropic glutamate receptors, are ubiquitously expressed in the CNS and play a pivotal role in learning and memory. Accordingly, their function is tightly regulated by cellular signalling cascades that converge upon constituent subunits to alter NMDAR function through post-translational modifications. In keeping with this, signalling cascades recruited downstream of GPCRs can readily influence NMDAR function and in this way alter learning and memory. In addition to influencing learning and memory, GPCRs are also an important target in treating myriad psychiatric disorders, for example the serotonin and dopamine receptors represent important targets for antipsychotic drugs [8,9]. Likewise, dysregulated NMDAR function contributes to psychiatric illnesses as illustrated by the schizophrenic-like symptoms observed in humans upon administration of NMDAR antagonists, such as ketamine and phencyclidine (PCP). These and other findings have contributed to the development of the NMDAR hypofunction theory of schizophrenia, which has found increasing support in recent years [10]. To date, the development of therapeutically effective agents capable of directly modulating NMDAR function has met with limited success at best. However, as discussed in the following sections, the NMDAR likely represents an important downstream effector of GPCRs that have been targeted by established and emerging neuropsychiatric agents.

## 2. Introduction to NMDARs (NMDA Receptors)

NMDARs are tetramers composed of two GluN1 subunits and two GluN2 subunits or in some cases, a GluN2 and a GluN3 subunit [11]. Structurally, NMDAR subunits are composed of: (1) two flexible extracellular lobes; the *N*-terminal domain (NTD) and agonist-binding domain (ABD); (2) three transmembrane segments and a re-entrant loop; and (3) a *C*-terminal tail that interacts with various intracellular proteins [12]. The NTD of NMDAR subunits plays an important role in subunit assembly [13]. In GluN2A and GluN2B subunits, this region represents the binding site for allosteric inhibitors such as Zn^2+^ and Ro25-6981 respectively [14,15]. Of note, opening of the NMDAR channel requires binding of not only glutamate, but also glycine (co-agonist) to GluN2 and GluN1 subunits, respectively. When the agonists bind, they stabilize a closed-cleft conformation of the two extracellular lobes (NBD and ABD) which causes the receptor channel to open. In contrast, competitive antagonists bind the same domains but impede cleft closure and prevent channel activation [16].

### 2.1. GluN1 Subunits

GluN1 is expressed ubiquitously in the brain. Its gene (*Grin1*) consists of 22 exons and alternative splicing of three of these (exons 5, 21 and 22) generates eight different isoforms [17]. Exon 5 encodes a splice cassette within the extracellular *N*-terminus (termed N1), whereas exons 21 and 22 encode two splice cassettes within the intracellular *C*-terminus of the GluN1 subunit (termed C1 and C2 respectively) [17]. The splicing of the C2 cassette removes the first stop codon and encodes a different cassette (termed C2′) [17]. GluN1 subunits do not form functional receptors alone. When expressed in the absence of GluN2 subunits, GluN1 isoforms containing N1, C1 and C2 cassettes are retained in the ER [18], due to the presence within the C1 cassette of a ER retention motif [19]. In contrast, when co-expressed with GluN2 subunits the ER retention motif is masked allowing for the release of GluN1/GluN2 receptors from ER and trafficking to the cell surface [19]. In addition, the splice status of GluN1 can influence the functional modulation of NMDARs by protein kinase A (PKA) and protein kinase C (PKC). Consensus serine residues within the C1 cassette of GluN1 subunit are phosphorylated by PKA and PKC [20,21]. Interestingly, PKC phosphorylation within C1 relieves ER retention and enhances GluN1 surface expression [22].

### 2.2. GluN2 Subunits

The family of GluN2 subunits consists of GluN2A, GluN2B, GluN2C and GluN2D. GluN2A and GluN2B subunits are the predominant subunit in higher brain structures [23]. GluN2C subunits is highly expressed in the cerebellum while the expression of GluN2D subunits is mainly restricted to the brainstem [24]. During development, the expression of GluN2B and GluN2D subunits is abundant and then decreases during maturation. Conversely, expression of GluN2A and GluN2C subunits is low during development and then increases during maturation [11]. At mature synapses in the hippocampus, GluN2A subunits predominate at the synapse whereas GluN2B subunits predominate at extrasynaptic sites [23]. This differential subcellular distribution has important functional consequences as will be discussed in the following sections.

#### 2.2.1. Intracellular Association of GluN2 Subunits

At synaptic sites, PDZ-binding motifs (conserved amino acid sequence ESDV) within the distal *C*-terminus of both GluN2A and GluN2B subunits contribute to subunit retention by interacting with the membrane-associated guanylate kinase (MAGUK) family of synaptic scaffolding proteins. These include postsynaptic density protein 95 (PSD-95), postsynaptic density 93 (PSD-93), synapse-associated protein 97 (SAP97) and synapse-associated protein 102 (SAP102) [25]. Although it was initially suggested that GluN2A subunits selectively bound to PSD95 while GluN2B subunits preferentially interacted with SAP102 [26], more recently di-heteromeric GluN1/GluN2A receptors and GluN1/GluN2B receptors have been shown to interact with both PSD95 and SAP102 at comparable levels [27]. However, the interaction between GluN2 subunits (GluN2A and GluN2B) and MAGUK proteins can be differentially regulated through posttranslational modifications. Indeed, phosphorylation of GluN2B by casein kinase II (CK2) at S1480 of its PDZ binding motif disrupts the association of GluN2B with PSD-95 [28]. Moreover, CK2 phosphorylation of GluN2B, but not GluN2A, reduces the synaptic localization of GluN2B through increased endocytosis [29]. In the case of GluN2A, CaMKII phosphorylation of PSD-95 at Ser73, rather than of GluN2A itself, has been shown to disrupt the interaction between these two proteins [30].

In addition to regulation by serine/threonine kinases, phosphorylation by tyrosine kinases can influence the synaptic localization of GluN2 subunits by altering intracellular protein associations. For example, tyrosine phosphorylation of GluN2B regulates its interaction with the AP-2 adaptor, a protein complex mediating clathrin-mediated endocytosis. Specifically, tyrosine phosphorylation of GluN2B at Y_1472_ by Fyn disrupted its interaction with AP-2, thereby inhibiting endocytosis of GluN2B [31]. Conversely, tyrosine phosphorylation may prevent GluN2A removal from synaptic membranes by increasing their association with PSD-95 and thus protecting the subunits against degradation from calpain at a preferred cleavage site (residues 1278–1279) [32,33].

The differential association of NMDAR subunits with intracellular signalling proteins can also direct the contribution of GluN2A and GluN2B to different forms of synaptic plasticity. For example, CaMKII binds to GluN2B subunits with high affinity whereas the interaction with GluN2A is weak [34]. This is reflected in the finding that when CaMKII is activated by CaM, it relocates to the synapses where it strongly associates with GluN2B [34]. Importantly, the interaction with GluN2B can lock CaMKII in an autonomous, constitutively active state that functions independently of Ca^2+^/CaM [35]. Another interesting protein interaction at the synapses occurs between GluN2B subunits and Ras protein-specific guanine nucleotide-releasing factor 1 (RasGRF1), a CaM dependent Ras guanine nucleotide releasing factor. This interaction has been proposed to facilitate ERK activation [36].

#### 2.2.2. Distinct Functional Roles of GluN2 Subunits in Synaptic Plasticity

Collectively, the findings from studies summarized here and many others have firmly established that the various GluN2 subunits have distinct regional expression profiles that vary with developmental stage, differ in their biophysical and pharmacological profiles, generate distinct Ca^2+^ signals and are differentially regulated by biochemical pathways contributed via distinct interactions with signalling partners. More difficult to reconcile is the specific physiological functions contributed by each of the heterogeneous GluN2-containing NMDAR subpopulations. This question is especially significant when considering GluN2A and GluN2B, the two major GluN2 subunits with overlapping expression in the CNS. More recently, a resolution to this apparent conundrum appeared at hand when it was suggested that GluN2ARs are required for the induction of LTP (long term potentiation) while GluN2BRs are responsible for LTD (long term depression) induction [37,38]. This proposal immediately raised considerable controversy; three research groups subsequently demonstrated that blocking GluN1/GluN2BRs did not prevent the induction of LTD [39]. Another study even suggested that the GluN2BR antagonist ifenprodil enhanced the induction of LTD in the CA1 region of the hippocampus [40], suggesting that GluN2BRs are functionally opposed to the induction of this form of plasticity. Conversely, other electrophysiological studies have shown that GluN2BR activation can in fact promote the induction of LTP induced by a variety of stimulation protocols [41]. For example, as discussed earlier, GluN2B can mediate LTP by directly associating with CaMKII [42]. In addition, studies in transgenic animals have shown that LTP can still be induced in GluN2A subunit knockout mice. Similarly, mice overexpressing GluN2B have enhanced LTP [43,44]. Accordingly, a clear functional segregation between LTD/LTP induction based on NMDAR subunit composition alone is difficult to reconcile with accumulated evidence.

More likely, as GluN2ARs and GluN2BRs are activated in concert when synaptic plasticity is induced experimentally, each receptor subpopulation contributes uniquely to the resulting rise in postsynaptic Ca^2+^. Moreover, their distinct biophysical properties dictate that the contribution of each receptor subtype will vary according to the stimulation patterns that promote their activation. For example, it is well recognized that GluN1/GluN2A receptor-mediated currents exhibit faster rise, desensitize more extensively and deactivate more rapidly than GluN2BRs [38]. On this basis, a kinetic model constructed from empirically derived GluN2AR and GluN2BR single-channel kinetics, predicts that GluN2BR signalling should predominate during low-frequency repetitive stimulation and conversely that charge transfer through GluN2ARs would exceed that of GluN2BRs during high-frequency long term potentiation (LTP) inducing stimulation [40]. In light of this, it is perhaps not surprising that the balance between GluN2AR and GluN2BR activation, and the consequent signalling cascades recruited, is increasingly viewed as a critical determinant governing the direction of synaptic plasticity.

#### 2.2.3. GluN2 Subunits in Metaplasticity

It is well known that the threshold for the induction of LTP and LTD can be influenced by prior activity. This plasticity of plasticity has been termed metaplasticity [45–47]. Conceptually, metaplasticity is best understood by considering the relation between neuronal activity and the induction of bidirectional synaptic plasticity as originally modelled by Bienenstock, Cooper and Munro (BCM model) [48]. Based upon observations of experience-dependent plasticity in the kitten visual cortex these authors proposed a modification threshold (θ_M_) for the induction of plasticity; LTP is induced when postsynaptic activity lies above this threshold and conversely, LTD is induced when the level falls below it. For example, in dark-reared kittens θ_M_ is reduced at excitatory synapse of the visual cortex reflecting a decrease in the threshold for LTP induction [49]. Metaplasticity has also been demonstrated in the hippocampus and the mechanisms responsible for setting the modification threshold for synaptic plasticity are emerging [50,51].

Most experimental protocols developed to investigate mechanisms of metaplasticity involve induced changes in neuronal activity prior to the induction of synaptic plasticity. Changes in neuronal activity have typically been induced in response to electrical, pharmacological or behavioral stimuli and the resulting metaplasticity is contingent upon the activation of NMDARs [45]. Moreover, metaplasticity has been shown to be associated with changes in NMDAR signalling, specifically the relative contribution of GluN2ARs and GluN2BRs to synaptic transmission [46–48]. Indeed, light deprivation decreases the ratio of GluN2AR/GluN2BR, as reflected by more slowly deactivating NMDAR currentsin layer 2/3 of visual cortex. In contrast, exposure to visual stimulation increased the ratio and induced more rapid NMDAR currents [46]. These changes in the ratio of GluN2AR/GluN2BR were accompanied by corresponding changes in the threshold for LTP/LTD induction [52]. In addition, in GluN2A^−/−^ mice metaplasticity in the visual cortex was lost [48]. Metaplasticity can also be induced by mild sleep deprivation (4–6 h), shown to selectively increase GluN2AR surface expression in adult mouse CA1 synapses and facilitate LTD induction at these synapses. Furthermore in the GluN2A^−/−^ mice, this form of metaplasticity is absent [53].

In addition to its regulation by behavioral stimuli, the ratio of GluN2AR/GluN2BR is also modulated by priming electrical stimulation. Priming stimulations across a wide range of frequencies (1–100 Hz) can alter the ratio of GluN2AR/GluN2BR, resulting in changes to LTP/LTD induction [50]. One mechanism to explain metaplasticity by priming stimulation is through altered tyrosine phosphorylation of NMDARs through SFKs (Src family kinases). Consequently, even if prior activity does not itself cause substantial NMDAR activation, such activity can nevertheless cause the activation of several GPCRs, which in turn regulate NMDAR function and thus the ability to subsequently induce plasticity [45]. Several GPCRs can regulate the function of NMDARs through SFKs [54,55] and in this way modify the threshold for the induction of LTD/LTP. Specifically, we recently reported that stimulation of selected GPCRs that enhance the function of GluN2ARs favors LTP over LTD, whereas the converse occurs with stimulation of distinct GPCRs that enhance the function of GluN2BRs [51]. Importantly, this does not exclude the possibility that both subtypes of receptors contribute to both forms of synaptic plasticity but rather, is consistent with evidence that dynamic changes in the ratio of GluN2ARs and GluN2BRs signalling provides a mechanism for metaplasticity.

#### 2.2.4. Tri-heteromeric GluN1/GluN2A/GluN2B Receptors in Synaptic Plasticity

Several studies have suggested that in addition to di-heteromeric NMDARs (GluN1, GluN1, GluN2x, GluN2x), tri-heteromeric NMDARs (GluN1, GluN1, GluN2x, GluN2y (or GluN3x)) may exist in some brain areas. Although the physiological role and pharmacological properties of di-heteromeric NMDAR are well studied, relatively little is known about tri-heteromeric NMDARs. Recent studies have suggested that tri-heteromeric NMDARs are predominantly expressed at synapses in adult hippocampus [56]. Tri-heteromeric NMDARs are reported to possess distinct pharmacological properties when compared to di-heteromeric NMDARs. Evidence suggests that tri-heteromeric GluN1/GluN2A/GluN2B receptors have an “intermediate” sensitivity to both GluN2AR and GluN2BR antagonists [15,57,58]. The hybrid nature of tri-heteromeric NMDARs raises intriguing possibilities regarding their role in synaptic plasticity. Indeed, LTP induction at mature synapses was suggested to require both di-heteromeric GluN1/GluN2A and tri-heteromeric GluN1/GluN2A/GluN2B receptors [59].

## 3. The Regulation of NMDARs by G Protein Coupled Receptor (GPCR)

All NMDAR subunits have large intracellular *C*-terminal tails that contain serine, threonine and tyrosine residues representing potential sites of phosphorylation, for example by protein kinase A (PKA), protein kinase C (PKC) and Src family kinases (SFKs) [60–62]. Phosphorylation at these sites regulates NMDAR channel activity through a variety of means including changes in single channel conductance, surface expression and receptor trafficking [60–62]. Accordingly, by recruiting these kinases to phosphorylate NMDAR subunits, GPCRs can regulate NMDAR expression and channel function at synaptic and extrasynaptic sites [63]. In the following sections we summarize recent work examining the regulation of NMDARs downstream of GPCRs. The intent is not to provide an exhaustive overview of all GPCRs shown to influence NMDAR function. Rather we focus on selected GPCRs that couple to each of the major classes of Gα subunits with an aim towards highlighting the rich variety of mechanisms through which NMDARs are regulated. A particular focus is on studies of the hippocampus where NMDARs and their contribution to synaptic plasticity have been extensively characterized. Nevertheless, in many instances the findings discussed are likely relevant to the function of NMDARs in other regions, at least conceptually. Notably, we highlight several instances where regionally divergent mechanisms have been reported for a given GPCR.

### 3.1. The Regulation of NMDARs by Gαq Containing GPCRs

Characteristically, the activation of Gαq containing GPCRs increases the activity of PKC. PKC is divided into three groups that include the conventional, novel and atypical PKC isoforms. The conventional PKCs are activated by Ca^2+^ and DAG while the novel PKCs, which lack a Ca^2+^ binding domain, are only stimulated by DAG. In contrast, the atypical PKCs are only sensitive to phospholipids; both Ca^2+^ and DAG fail to activate them. When PKC is activated, it will translocate to the membrane from the cytosol [64].

PKC activation can increase NMDAR mediated currents in both isolated and cultured hippocampal neurons [55]. Biochemical studies have shown that GluN1, GluN2A, GluN2B and GluN2C subunits can be phosphorylated by PKC *in vivo* and *in vitro* [21,65–67]. However, when the PKC phosphorylation sites of NMDAR are mutated to Ala, PKC still potentiates NMDAR currents, indicating that PKC signals through another molecule to regulate NMDAR currents [68]. Our laboratory demonstrated that this signalling molecule is Src. When Src inhibitory peptide (Src (40–58)) is applied in the patch pipette, PKC fails to increase NMDAR currents [55]. In addition, cell adhesion kinase β (CAKβ), which is a member of the focal adhesion kinase (FAK) family, was shown to work as an intermediary between PKC and Src to regulate NMDAR [69]. Via Src activation, PKC modulates channel activity, not only by changing physical properties of receptors, but also by regulating receptor trafficking via synaptosome-associated-protein receptor (SNARE) dependent exocytosis [70–72]. Furthermore, the interaction of NMDARs with PSD95 and SAP102 enhances the surface expression of NMDARs and occludes PKC potentiation of channel activity [70,73].

**Figure 1.**
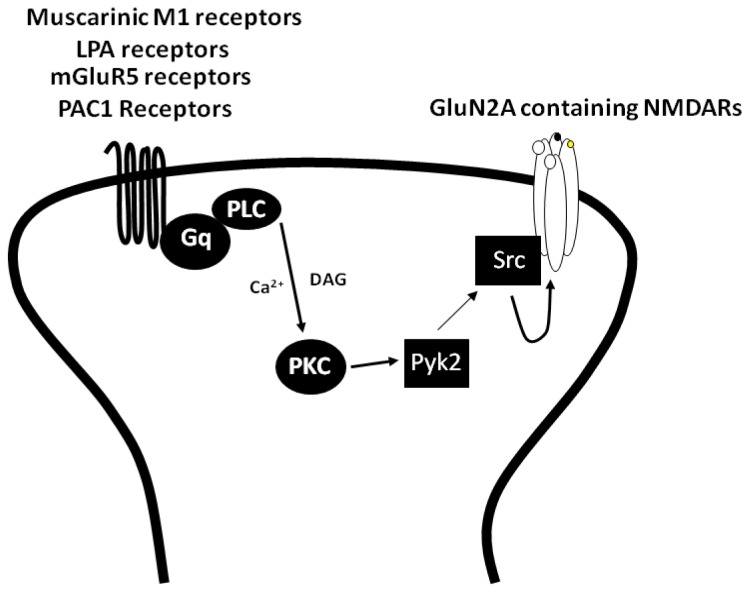
The activation of Gαq coupled receptors induces PLC/PKC/Pyk2/Src signalling to enhance GluN2A containing NMDAR function.

Not surprisingly, many Gαq coupled GPCRs can modulate NMDAR activity via PKC dependent pathway. In this regard, activation of the PAC1 receptors, which is coupled to Gαq proteins [74], increases NMDAR mediated currents via the PKC/CAKβ/Src signalling pathway [75] (Figure 1). Other Gαq coupled GPCRs including muscarinic M1, lysophosphatidic acid (LPA) and metabotropic glutamate receptor subtype 5 (mGluR5) have been shown to enhance NMDAR currents through this signalling pathway [54,55,76] (Figure 1). In addition, at hippocampal mossy fiber synapses, activation of postsynaptic adenosine A2A receptor (a Gαq coupled receptor) enhances NMDAR-mediated excitatory postsynaptic currents (EPSC_NMDA_) via G protein/Src pathway. Similarly, this pathway is proposed to be involved in the LTP of EPSC_NMDA_ induced by HFS [77]. At Schaffer collateral synapses acetylcholine (ACh) induces a long-lasting synaptic enhancement of EPSC_NMDA_. This action was mediated by M1 receptors and the activation of these receptors stimulated the PKC/Src signalling pathway to increase EPSC_NMDA_ [78].

A notable feature of the regulation of NMDARs by GPCRs acting through Gαq recruited pathways is that peak currents are enhanced to a greater extent than the steady-state of NMDA-evoked currents. In part, this can be attributed to a PKC-dependent increase in Ca^2+^-dependent inactivation and glycine-insensitive desensitization [79,80]. However, the preferential enhancement of peak NMDAR currents can also be attributed to the differential augmentation of GluN2AR *vs.* GluN2BR function by Gαq GPCRs. Indeed, due to kinetic differences between the activation rates of GluN2ARs and GluN2BRs, NMDA peak currents are more likely to be contributed by GluN2ARs, while GluN2BRs contribute more strongly to the sustained or steady-state component of the currents [81]. This led us to propose that PAC1 receptor activation, and more broadly signalling via PKC/Src, specifically targets GluN2A-containing NMDAR to increase NMDA-evoked currents (Figure 1). Three lines of evidence suggested that the activation of the PAC1 receptors preferentially increases the activity of GluN2ARs. Firstly, a GluN2AR preferring antagonist, NVP-AAM077, blocks NMDAR potentiation by the PAC1 receptors, whereas a GluN2BR selective antagonist, Ro25-6981, does not [51]. Secondly, Zn^2+^, a selective inhibitor of GluN2ARs at nanomolar concentrations [15,82], blocks the potentiation of NMDARs by the PAC1 receptors [51]. Finally, in GluN2A^−/−^ mice, the activation of the PAC1 receptors fails to increase NMDAR mediated currents [51]. More, recent evidence suggests that other Gαq-coupled GPCRs also selectively augment the function of GluN2A-containing NMDARs. Indeed, the application of orexin increased surface expression of GluN2ARs but not GluN2BRs in the VTA via OXR1 receptors/Gαq/PKC signalling [83]. Note however, that these studies did not demonstrate whether the differential regulation of GluN2ARs and GluN2BRs by these GPCRs requires SFK.

### 3.2. The Regulation of NMDAR by Gαs Containing GPCRs

Stimulation of Gαs containing GPCRs increases the concentration of cAMP and activates PKA, which consists of two catalytic subunits and two regulatory subunits. When cAMP binds to the regulatory subunits, PKA activity is increased. Pathways involving PKA are known to regulate NMDAR function, presumably via phosphorylation at sites identified on GluN1, GluN2A and GluN2B subunits [84]. For example, the Ca^2+^ permeability of NMDARs is under the control of the cAMP/PKA signalling cascade and PKA inhibitors can reduce the relative fraction of Ca^2+^ influx through NMDARs [85]. Moreover, by phosphorylating inhibitor-1, the activation of PKA inhibits protein phosphatase-1 and consequently enhances NMDAR channel activity through increased receptor phosphorylation [86]. Additionally, acting in concert with PKC, PKA phosphorylation within an ER retention motif located at the *C*-terminus of the GluN1 subunit releases GluN1 from the ER and increases the surface expression of NMDARs [87].

**Figure 2.**
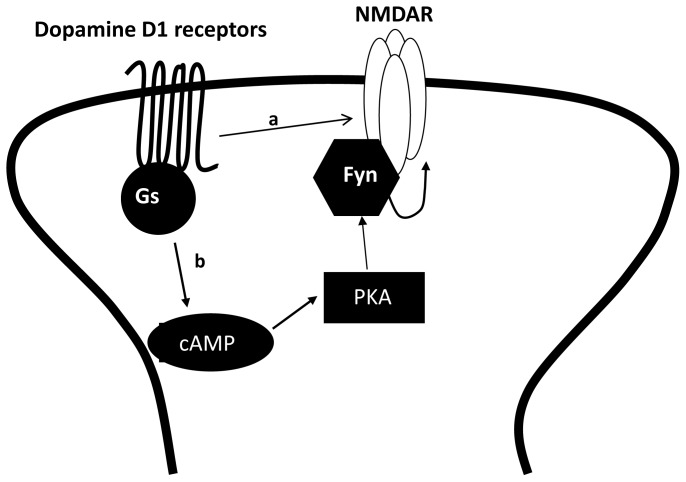
In the hippocampus, the regulation of NMDARs by Gαs coupled receptors involves multiple mechanisms. (**a**) Dopamine D1 receptors and NMDAR can communicate through direct physical interactions, which inhibits NMDAR activity; (**b**) in addition, dopamine D1 receptor activation can enhance NMDAR mediated currents through a PKA dependent signalling pathway.

The regulation of NMDARs by dopamine D1 receptor (D1R), a Gαs coupled receptor, has been extensively studied in different brain regions [88–90]. The most prominent mechanism through which D1R activation enhances NMDAR activities is via PKA-dependent mechanisms [91]. In the hippocampus, activation of D1Rs potentiates NMDAR-mediated responses through cAMP/PKA-dependent recruitment of a non-receptor tyrosine kinase of the Src family, specifically Fyn [51] (Figure 2). Indeed, the activation of D1Rs stimulates Fyn kinase activity in hippocampal slices [51] (Figure 2). More importantly, NMDAR potentiation by D1R stimulation is prevented by a selective Fyn inhibitory peptide. This potentiation is selective for GluN2B-containing NMDARs given that it can be inhibited by Ro 25-6981 but not by the GluN2A-preferring inhibitors, NVP-AAM077 or Zn^2+^. Additionally, genetic deletion of GluN2A subunits does not prevent the enhancement of NMDAR mediated currents downstream of D1Rs, confirming that GluN2A subunits are not required for this enhancement [51] (Figure 2). Potentiation of NMDAR function via cAMP/PKA/Fyn is also observed following the activation of VIP receptors, another Gαs coupled GPCR [51] (Figure 2).

The Fyn/GluN2BR dependence of potentiation by D1R is consistent with other studies [88,89,92]. For example, D1R activation increases surface expression of NMDARs in the striatum. Increased NMDAR surface expression in this context is contingent on Fyn kinase as it is not observed following D1R stimulation in Fyn^−/−^ mice [88,89]. Similarly, in cultured PFC neurons the activation of D1Rs increases the surface expression of GluN2B containing NMDARs [92]. More broadly, the dopamine D5 receptor, which is also coupled to Gαs and cAMP/PKA signalling, has been shown to recruit GluN2BRs from the cytosol to synaptic sites and thereby potentiate NMDAR currents [93]. However, whether the differential regulation of GluN2ARs and GluN2BRs by the D5 receptor also requires Fyn kinase was not tested in this study. Additionally, dopamine D1/5 receptor stimulation has been shown to enhance LTP through PKA-dependent enhancement of SFK activity and GluN2BR function [94].

Importantly, D1R are also capable of regulating NMDAR function independently of PKA-dependent signalling through direct physical coupling. Direct protein-protein interactions were identified between the *C*-terminal tails of the dopamine D1R and either the GluN1 subunit or GluN2A subunit of NMDAR [84]. The interaction of dopamine D1R with the GluN2A subunit suppresses NMDAR currents by decreasing the surface expression of NMDARs. This effect is entirely independent of PKA and PKC signalling cascades [84]. Functionally, the interaction between D1R and the GluN1 subunit protects neurons from NMDA-mediated cell death [84]. In addition, a peptide that disrupts the interaction of the D1R-NMDAR complex inhibits NMDAR dependent LTP and induces working memory deficits [85].

As highlighted by these numerous studies, D1Rs can regulate NMDAR function through multiple molecular mechanisms. Functionally, the regulation of NMDARs by D1Rs through second messenger systems may be countered by the effects mediated through the direct physical interaction between these two receptors. Thus, direct D1R-NMDAR interactions may serve as a molecular brake on the augmentation of NMDARs via the D1R mediated PKA pathway. Importantly, it remains unclear the degree to which these distinct mechanisms overlap and if so, the mechanisms that govern the resulting functional outcomes.

### 3.3. The Regulation of NMDAR by Gαi Containing GPCRs

**Figure 3.**
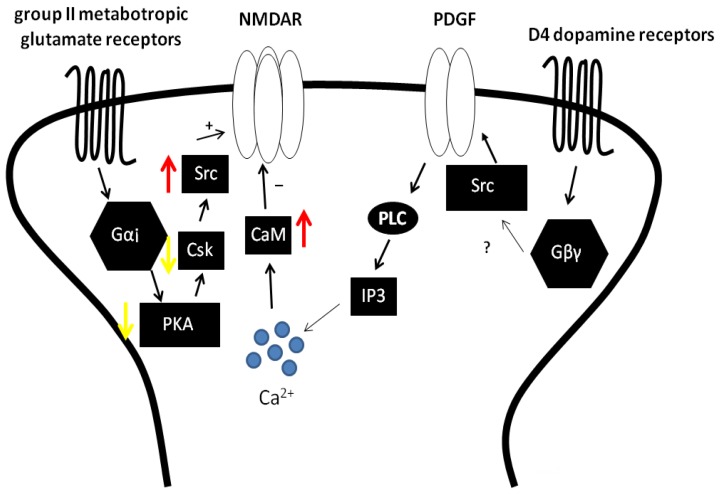
Gαi coupled receptors increase or decrease NMDAR function depending on the signalling pathways recruited. The activation of dopamine D4 receptor depresses NMDAR mediated currents by transactivating PDGF receptors. The resulting increased PLC activity hydrolyzes PIP_2_ leading to the generation of IP_3_ and DAG. The release of Ca^2+^ from internal stores following IP_3_ receptor stimulation activates CaM, which then promotes calcium dependent inactivation of NMDARs. In contrast, activation of group II mGluRs enhance NMDAR mediated currents via PKA/Csk/Src dependent pathway.

In contrast to Gαs coupled GPCRs, the activation of Gαi coupled receptors characteristically reduces the concentration of cAMP and inhibits PKA activity. But their activation also induces other signalling pathways which are independent of PKA. Accordingly, they may potentiate or depress NMDAR function depending on the signalling pathways recruited [54,95] (Figure 3).

In the hippocampus, the activation of dopamine D4 receptor (D4R), which couples to Gαi, depresses NMDAR mediated currents (Figure 3). Surprisingly, this response is not mediated by the inhibition of PKA [96]. Rather, platelet-derived growth factor receptors (PDGF-Rs) are involved as shown by evidence demonstrating that the depression of NMDAR currents following D4R activation is prevented by PDGF-R inhibitors and can be occluded following PDGF-R activation by application of PDGF [97]. Mechanistically, D4 receptors transactivate PDGF-Rs and depress NMDAR function in a PLC, but not PKC, dependent manner (Figure 3). Moreover, the depression of NMDAR currents is blocked by calmodulin (CaM) binding-peptides and occluded when cells are treated with CaM [96], consistent with increased Ca^2+^-dependent inactivation as a result of D4R-PDGF-R signalling. Indeed, Ca^2+^-CaM is known to compete with actin for binding to the C0 domain of the NR1 subunit, promoting calcium dependent inactivation of NMDARs [98,99]. Accordingly, it is proposed that PDGF receptors are transactivated by D4Rs, as a result of which PLC activity is increased, resulting in increased levels of IP_3_ and DAG. IP_3_ binds to IP_3_ receptor and stimulates Ca^2+^ released from ER (Figure 3), which ultimately causes increased Ca^2+^-dependent inactivation of NMDARs. Interestingly, a previous study has shown that PDGF can depress NMDAR currents in a PKA dependent manner via the PDGF-R [100]. In contrast, D4R-PDGFR-mediated depression does not require PKA [96]. These findings suggest that signalling downstream of PDGFRs in the hippocampus is contingent on the manner in which these receptors become active.

Dopamine receptor regulation of NMDARs has been investigated in other regions of the CNS where diverse signalling mechanisms have been reported. For example, in pyramidal neurons from the prefrontal cortex (PFC), transactivation of PDGF-Rs in response to the application of quipirole, a D2-class dopamine receptor agonist, has also been shown to regulate NMDAR function. However, in this instance, the D2R rather than the D4R has been implicated [101]. The resulting inhibition may involve reduced NMDAR surface expression [102] through inhibition of PKA and subsequent protein phosphatase 1 (PP1)-mediated inhibition of CaMKII [102]. In the striatum, physical coupling with dopamine receptors has been implicated in the regulation of NMDARs. Indeed, dopamine stimulation by cocaine enhances the physical association between D2 receptors (D2Rs) and GluN2B containing NMDARs in striatum [103]. Increased D2R-GluN2B interaction stimulated by cocaine interferes with the binding of CaMKII to GluN2B, which reduces GluN2B phosphorylation at Ser1303, leading to inhibition of NMDAR function [103].

In contrast to the depression of NMDAR function by D4R and D2R, the activation of distinct Gαi coupled GPCRs have been reported to potentiate NMDAR function in the hippocampus (Figure 3). For example, the activation of group II mGluRs (Gαi/o protein coupled receptors) enhances NMDAR mediated currents via a PKA-and Src-dependent pathway that selectively targets GluN2A-containing NMDARs [95] (Figure 3). This Gαi/o-mediated activation of Src differed from that of Gαq-coupled receptors that signal through PKC and CAKβ [54,55,75]. Rather, Group II mGluRs receptors activate Src kinase by inhibiting cAMP/PKA signalling and consequently the activity of *C*-terminal Src kinase (Csk), a negative regulator of Src activity [95] (Figure 3). The ability of PKA to negatively regulate NMDAR function through Csk-mediated inhibition of Src activity was established in a previous study where the catalytic fragment of PKA was shown to inhibit Src-mediated potentiation of NMDARs in inside-out patches from cultured hippocampal neurons [100]. As with dopamine receptors, the mechanisms underlying the regulation of NMDARs by group II mGluRs varies regionally. In prefrontal cortex, group II mGluRs signal through PKC to enhance NMDAR activities [104]. Both SNARE (Soluble *N*-ethylmaleimide-sensitive factor activating protein receptor) and Rab4 have been reported to contribute to the group II mGluR-induced enhancement of NMDAR currents [97]. Given that the function of Rab4 and SNARE proteins can be regulated by PKC [72], group II mGluRs may enhance the SNARE-mediated NMDAR exocytosis through PKC signalling.

### 3.4. The Regulation of NMDAR by Gα_12/13_ Containing GPCRs

**Figure 4.**
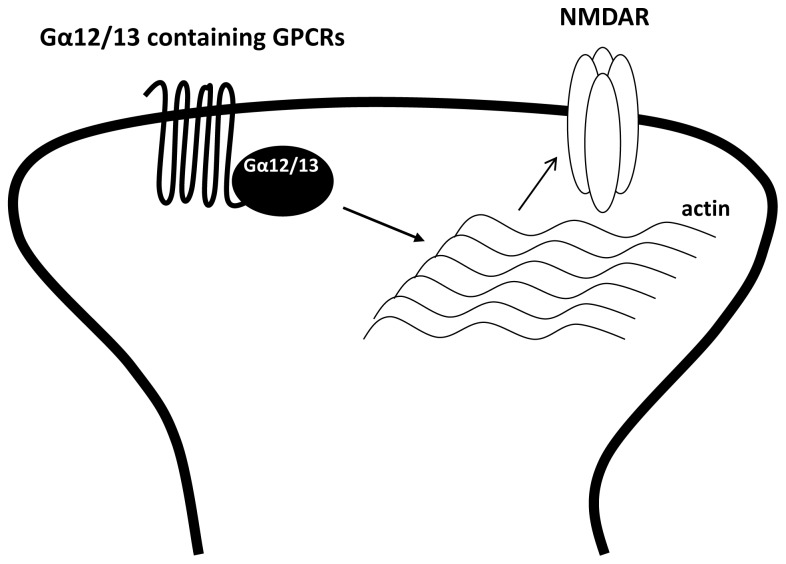
Gα_12/13_ coupled GPCRs can modulate NMDARs indirectly via their effects on actin cytoskeleton dynamics.

It is well established that Gα_12_ and Gα_13_ regulate the activity of small GTPase Rho through guanine nucleotide exchange factor (RhoGEF) and modulate various cellular responses [105]. In addition to RhoGEF, Gα_12_ and Gα_13_ can also regulate the actin cytoskeleton and myosin activity [105]. NMDARs are known to associate with the cytoskeleton via protein–protein interactions [98,99,106]. Accordingly, actin cytoskeleton dynamics are an important determinant of NMDAR function. GluN1 and GluN2 subunits of NMDARs couple to the actin cytoskeleton via the actin binding proteins actinin2 and spectrin respectively [99]. Through these interactions with the cytoskeleton, NMDAR function is influenced by changes in actin cytoskeleton integrity (Figure 4). This is exemplified by experiments showing that actin depolymerization reduces NMDAR function. Moreover, the influx of Ca^2+^ associated with strong NMDAR activation has been shown to disrupt the interaction of NMDARs with the cytoskeleton, through a Ca^2+^-dependent mechanism, causing an irreversible downregulation of NMDAR activity [107].

Myosin is also associated with NMDARs. Constitutively active myosin light chain kinase (MLCK) enhances NMDAR-mediated currents in both acutely isolated CA1 pyramidal and cultured hippocampal neurons, whereas inhibitors of MLCK depress these currents [108]. These effects of MLCK require an intact cytoskeleton as both MLCK inhibitors and constitutively active MLCK are ineffective when applied to neurons pretreated with latrunculin B, a drug that induces actin filament depolymerization [108]. It is proposed that MLCK might activate myosin and cause physical tension to be transmitted via actin to the NMDARs. Alternatively, it may alter the physical relation between the actin cytoskeleton and NMDARs, resulting in the modification of NMDAR activity.

An increasing number of GPCRs are reported to couple through Gα_12/13_ proteins, including purinergic receptors (P_2_Y_1_, P_2_Y_2_), muscarinic acetylcholine receptor (M1 and M3), serotonin (5-HT_2_C and 5-HT_4_), and many more are likely and waiting for identification [109]. The activation of 5-HT_1_A selectively inhibits GluN2B containing NMDARs via a microtubule-dependent mechanism [110]. Drugs that interfere with microtubules assembly blocked this inhibition. In addition, knock-down of the kinesin motor protein KIF 17 (kinesin superfamily member 17), which transports GluN2B-containing vesicles along microtubule in neuronal dendrites, also prevents 5-HT_1_A induced inhibition of NMDAR [110]. But whether Gα_12/13_ proteins are involved was not investigated.

## 4. Conclusions

As discussed, the regulation of NMDARs by GPCRs involves numerous intracellular signalling molecules and regulatory proteins. Moreover, increasing evidence suggests that specific assemblies of NMDAR subunits are selectively targeted downstream of a given GPCR. The complexity of processes regulating NMDARs is increased by regional variations in the mechanisms and functional outcomes observed following the activation of a given GPCR. Recognizing that the NMDAR is an important downstream effector of GPCRs has implications for understanding processes that modulate learning and memory, contribute to neurological disorders in which GPCR signalling is altered and influence treatment outcome for therapeutic agents that act upon these same receptors. The pathology and treatment of schizophrenia represents a notable example. Schizophrenia is a complex psychiatric disorder with a strong genetic component. The clinical phenomena associated with schizophrenia can be grouped into positive symptoms (delusions, hallucinations, thought disorder), negative symptoms (anhedonia, blunted affect, social withdrawal), and cognitive deficits (inattention, executive function, and working memory) [111]. The long standing hyperdopaminergic hypothesis of schizophrenia remains a leading theory explaining the neurochemical basis of disease and current therapies for the treatment of psychosis (positive symptoms) focus on blockade of the dopamine D2 receptors [112,113]. However, numerous clinical and preclinical studies have led to the hypothesis that hypofunctional NMDARs may also play an important role in the pathophysiology underlying schizophrenia [114,115]. Importantly, these two theories may not be opposed in light of studies highlighted herein, showing that D2/D4R receptor stimulation provokes reduced NMDAR function in the hippocampus and cortex [96,101,102]. Accordingly, restoration of NMDAR function may represent a beneficial consequence of treatment with antipsychotic D2R blockers. As enhancing NMDAR function reduces the symptoms associated with schizophrenia [116], additional opportunities to provide beneficial outcomes in schizophrenia via stimulation of GPCRs that modulate NMDAR function are being sought. For example, selective mGlu5 receptor activation reduces both psychotic as well as negative symptoms and provides beneficial pro-cognitive activity [114]. Beyond schizophrenia, aberrant NMDAR function has been implicated in numerous neurological and neuropsychiatric disorders including Alzheimer’s disease [23,72], drug addiction [23,72], major depressive disorder [117] and anxiety disorders [118], to name but a few. Accordingly, adjusting the activity of NMDARs by targeting selected GPCRs may represent an attractive strategy in treating several neurological diseases.
